# Genetic variability of spelt factor gene in *Triticum* and *Aegilops* species

**DOI:** 10.1186/s12870-020-02536-8

**Published:** 2020-10-14

**Authors:** Valeriya Vavilova, Irina Konopatskaia, Alexandr Blinov, Elena Ya. Kondratenko, Yuliya V. Kruchinina, Nikolay P. Goncharov

**Affiliations:** Institute of Cytology and Genetics SB RAS, Novosibirsk, Russian Federation

**Keywords:** Spike morphology, Threshability, Spelt, Rachis fragility, *Q* gene, Evolution, *Triticum*, *Aegilops*

## Abstract

**Background:**

Threshability, rachis fragility and spike shape are critical traits for the domestication and evolution of wheat, determining the crop yield and efficiency of the harvest. Spelt factor gene *Q* controls a wide range of domestication-related traits in polyploid wheats, including those mentioned above. The main goal of the present study was to characterise the *Q* gene for uninvestigated accessions of wheats, including four endemics, and *Aegilops* accessions, and to analyze the species evolution based on differences in *Q* gene sequences.

**Results:**

We have studied the spike morphology for 15 accessions of wheat species, including four endemics, namely *Triticum macha*, *T. tibetanum*, *T. aestivum* ssp. *petropavlovskyi* and *T. spelta* ssp. *yunnanense*, and 24 *Aegilops* accessions, which are donors of B and D genomes for polyploid wheat. The *Q-5A*, *q-5D* and *q-5S* genes were investigated, and a novel allele of the *Q-5A* gene was found in accessions of *T. tibetanum* (KU510 and KU515). This allele was similar to the *Q* allele of *T. aestivum* cv. Chinese Spring but had an insertion 161 bp in length within exon 5. This insertion led to a frameshift and premature stop codon formation. Thus, the *T. tibetanum* have spelt spikes, which is probably determined by the gene *Tg*, rather than *Q.* We determined the variability within the *q-5D* genes among hexaploid wheat and their D genome donor *Aegilops tauschii.* Moreover, we studied the accessions C21–5129, KU-2074, and K-1100 of *Ae. tauschii* ssp. *strangulata*, which could be involved in the origin of hexaploid wheats.

**Conclusions:**

The variability and phylogenetic relationships of the *Q* gene sequences studied allowed us to clarify the relationships between species of the genus *Triticum* and to predict the donor of the D genome among the *Ae. tauschii* accessions. *Ae. tauschii* ssp. *strangulata* accessions C21–5129, KU-2074 and K-1100 are the most interesting among the analysed accessions, since their partial sequence of *q-5D* is identical to the *q-5D* of *T. aestivum* cv. Chinese Spring. This result indicates that the donor is *Ae. tauschii* ssp. *strangulata* but not *Ae. tauschii* ssp*. tauschii.* Our analysis allowed us to clarify the phylogenetic relationships in the genus *Triticum*.

## Background

Spike traits are critical for domestication, as they determine the crop yield and the efficiency of the harvest. At least four different loci are involved in control of spike traits in wheat species, namely, spelt factor gene *Q* (threshability, rachis fragility and spike shape), *non-brittle rachis 1* (*Btr1 -* spike fragility and its severity), *tenacious glumes* locus (*Tg –* threshability and rachis fragility) and *soft glume locus* (*sog* - threshability) [1–9]. *Q* gene located on the long arm of the chromosome 5A controls a wide range of domestication-related traits in polyploid wheat. Molecular cloning allowed the *Q* gene to be referred to the *APETALA2* (*AP2*)-like transcription factors and allowed two functional alleles *Q-5A* and *q-5A* to be described [1].

Most of the analysed cultivated wheat species are characterized by free-threshing, normal (or compact) spikes with non-fragile rachis and the presence of allele *Q-5A*. In contrast, the allele *q-5A* has been described in wild wheats with non free-threshing fragile spelt spikes [1, 2, 10]. Two single-nucleotide polymorphisms (SNPs) were described for the *Q* and *q* alleles: (1) G to C transition within exon 8 close to the AP2 domain regions, which results in non-synonymous substitution from valine to isoleucine and (2) neutral C to T substitution in the miRNA172 binding site within exon 10 [1–4, 11].

The regulatory mechanisms of *Q* gene expression remain poorly investigated. Based on the results of a two-hybrid yeast analysis, Simons et al. (2006) suggested that the presence of isoleucine at the 329 position, led to effective homodimer formation, which increases the *Q-5A* expression (1). On the other hand, it was shown that plant miRNA172 plays important role in floral development and regulation of *AP2*-like transcription factors [10, 12–15]. In recent studies, a great deal of attention has been paid to the role of miRNA172 in the regulation of the *Q* gene, since the variation in the miRNA172 target site increases the *Q* expression [16–18]. Thus, in mutants of the bread wheat cultivar Sunstate, Greenwood et al. (2017) described the allele *Q’*, which possesses the additional SNP within the miRNA172 binding site. The mutant plants showed a reduced height, compact spike phenotype and higher expression of the *Q’* allele (compared to the *Q* allele), due to the reduced level of miRNA172-dependent mRNA degradation, since miRNA172 cleaves the *q* allele transcripts more efficiently than those of *Q* and the reduced activity of miRNA172 causes spike compactness and easier threshing. Moreover, it was shown that overexpression of miRNA172 in transgenic wheats led to the formation of non-free-threshing (naked) grains and elongated spikes with an increased number of florets per spike [16]. Liu et al. (2018) showed that the *Q* gene is a nuclear transcriptional repressor which interacts with co-repressor TOPLESS, and they suggested that in *Triticum aestivum* L., *Q* expression is regulated by both miRNA172 and TOPLESS.

Despite the fact that *q-5B* represents a pseudogene and *q-5D* is expressed at a lower level compared to *Q-5A*, they both contribute to the suppression of spelt spike formation [2]. Recently a new allele *q’-5D* with a point mutation within the miRNA172 binding site, was identified [19]. The allele is characterized by an increased transcription level and pleiotropic effects on the spike compactness and plant dwarfness in *T. aestivum* cv. NAUH164. However, the *Q* gene is poorly investigated in *Aegilops* species, which are considered to be the donors of B and D genomes for polyploid wheat.

In the present study, *Q* genes were investigated from accessions of wheat species (including four endemic species, namely, *T. macha* Decapr. et Menabde, *T. tibetanum* Shao, *T. aestivum* ssp. *petropavlovskyi* (Udacz. et Migusch.) N.P. Gontsch. and *T. spelta* ssp. *yunnanense* (King ex S.L. Chen) N.P. Gontsch.), *Aegilops speltoides* Tausch and *Ae. tauschii* Coss. Based on variability and the phylogenetic relationships of *Q* gene sequences, we suggest the scheme of *Triticum* L. and *Aegilops* L. evolution.

## Results

### *Q* gene alleles in genus *Triticum* species

**Fig. 1:**
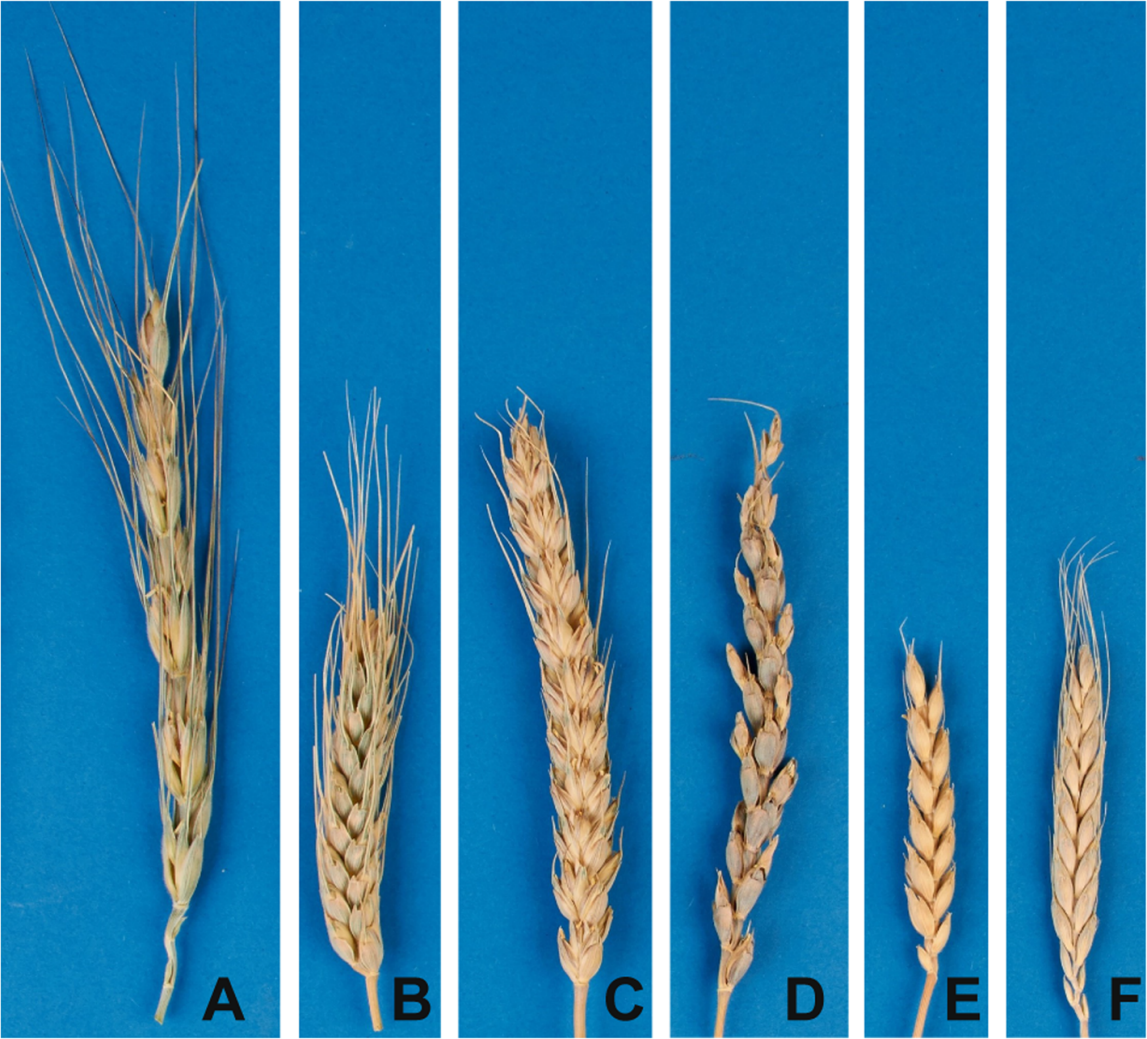
Spike morphologies of wheat species. **a** *T. aestivum* ssp. *petropavlovskyi* K-43351 (free-threshing, non fragile, spelt-like). **b** *T. spelta* ssp. *yunnanense* KU506 (non free-threshing, non fragile, spelt). **c** *T. spelta* ssp. *yunnanense* KU509 (non free-threshing, non fragile, spelt). **d** *T. vavilovii* Tri4630 (non free-threshing, non fragile, spelt). **e** *T. tibetanum* KU510 (non free-threshing, fragile (second type), spelt). **f** *T. macha* K-31689 (non free-threshing, fragile (second type), spelt)

In the present study, we determined three spike morphology traits for 15 accessions of wheat species including diploids (*T. monococcum* L., *T. urartu* Thum. ex Gandil., *T. boeoticum* Boiss.) and endemic hexaploids (*T. macha* Decapr. et Menabde, *T. aestivum* ssp. *petropavlovskyi* (Udacz. et Migusch.) N.P. Gontsch., *T. spelta* ssp. *yunnanense* (King ex S.L. Chen) N.P. Gontsch., *T. vavilovii* (Thum.) Jakibz., *T. tibetanum* Shao) (Fig. 1, Additional file 1: Table S1).

**Fig. 2:**
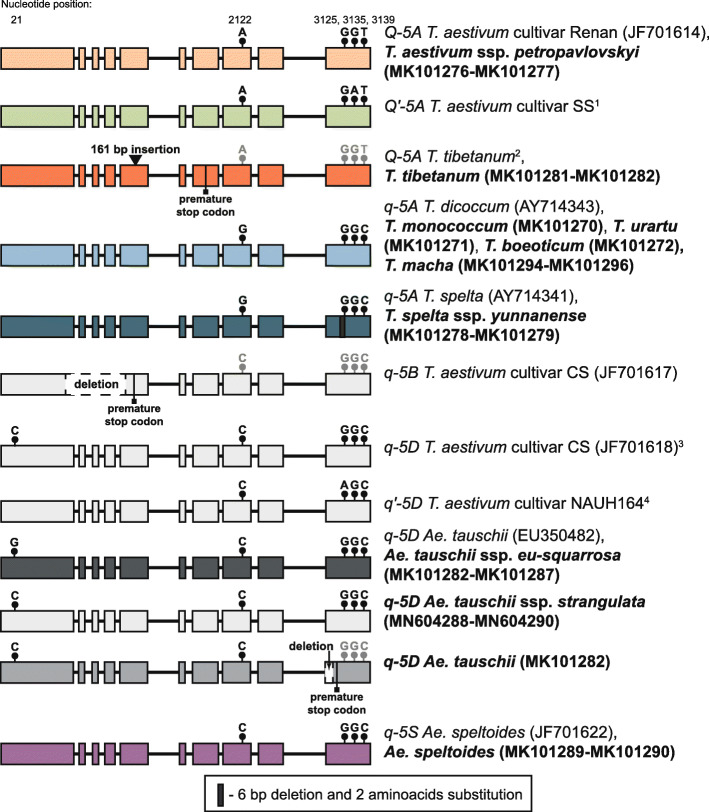
Scheme of *Q* gene alleles variability from various *Triticum* and *Aegilops* species. The numbers of nucleotides upstream from the start codon are given in accordance with the sequence *Q-5A* for *T. aestivum* (JF701619). Different A genome alleles are marked by different colours. Insertion is indicated by a black triangle. Single nucleotide polymorphisms are indicated by black circles. [1]- The *Q’-5A* allele was described by Greenwood et al. (2017); [2]- the *Q*^*t*^ allele was described in the present study in parallel with the study by Jiang et al. (2019); [3]- and *T. macha* (MK101294-MK101296), *T. tibetanum* (MK101301-MK101302), *T. spelta* ssp. *yunnanense* (MK101299-MK101230), *T. vavilovii* (MK101291-MK101293), *T. aestivum* ssp. *petropavlovskyi* (MK101297-MK101298); [4]- The *q’-5D* allele was described by Zhao et al. (2018)

We amplified full-length sequences of the *Q*-*5A* gene in three diploid and four hexaploid wheat species. The obtained sequences were compared with known *Q* and *q* alleles of wheat species available from GenBank. Comparative analysis and the following phylogenetic analysis showed that all diploid accessions and *T. macha* accessions with non free-threshing fragile spelt spikes had allele *5Aq*, identical to *T. dicoccum* (AY714343) (Fig. 2).

Sequences of *Q-5A* gene from two accessions of *T. aestivum* ssp. *petropavlovskyi* with free-threshing non fragile spelt-like spikes were identical to the allele *Q-5A* from *T. aestivum* cv. Renan (JF701614). The allele *q* of *T. spelta* (AY714341) was determined in accessions of *T. spelta* ssp. *yunnanense*, which were characterized by non free-threshing non fragile spelt spikes.

A novel allele of the *Q-5A* gene was described for *T. tibetanum* (KU510 and KU515) (Fig. 2, Additional file 1: Table S1). The allele was similar to the *Q* allele of *T. aestivum* cv. Chinese Spring, excluding an insertion 161 bp in length within exon 5 (Fig. 2). This insertion led to a frameshift and premature stop codon formation. In parallel, Jiang et al. (2019) described this allele for accessions of *T. tibetanum* and designated it *Q*^*t*^. The transposon insertion has led to re-acquisition of a wild trait (brittle rachises) in *T. tibetanum* [20]. No miRNA binding sites were predicted for the insertion region by psRNATarget.

We did not find the allele *Q’-5A* previously described for the *T. aestivum* cv. Sunstate mutant, among the analysed accessions [17].

We obtained no positive results from the PCR amplifications of *Q-5A* in the analysed accessions of *T. vavilovii* (Additional file 1: Table S1). The primers used in the present study allowed us to amplify only the full-length *q-5D* gene of this species. Comparative and phylogenetic analysis confirmed that the studied *T. vavilovii* accessions possess an allele of the *q-5D* gene identical to that of *T. aestivum* cv. Chinese Spring (Additional file 1: Table S3).

*T. macha*, *T. aestivum* ssp. *petropavlovskyi*, *T. spelta* ssp. *yunnanense*, and *T. tibetanum*, are characterized by the presence of allele *q-5D*, while the *q’-5D* allele was not found in any of the analysed wheat species (Fig. 2, Additional file 1: Table S1). Comparison of the *q-5D* gene with those of *T. aestivum* revealed species-specific and accession-specific substitutions within the introns (Additional file 1: Table S3). Two accessions possess the substitutions within the 10th exon. The *Q-5D* gene of *T. aestivum* ssp. *petropavlovskyi* is characterized by two non-synonymous substitutions, leading to the 397Ala- > Val and 405Ser- > Pro mutations. The synonymous substitution is presented in the position 3098 (G- > T) of the *Q-5D* of *T. vavilovii* (Additional file 1: Table S3).

### *Q* gene alleles in *Ae*. *tauschii* and *Ae.speltoides*

In the present study, we determined the spike morphology traits for 24 accessions of *Aegilops* species, which are donors of B and D genomes for polyploid wheat (Additional file 1: Table S4). Analysed accessions of *Ae. tauschii* were characterized by one of the following phenotypes: non-free-threshing fragile spelt or free-threshing fragile spelt (mutant accession TQ27) (Additional file 1: Table S4).

Full-length *q-5D* gene sequences were obtained for ten *Ae. tauschii* accessions (MK101282-MK101288, MN604288-MN604290). These sequences were compared with known *q-5D* sequences from *Ae. tauschii* and hexaploid wheat, including those identified in the present study. The sequences of the *q-5D* gene’s exons are highly conservative and the differences between the analysed sequences are limited to the introns. However, we have described a new allele with an SNP in position 72 and a 12-bp deletion affecting intron 9 and exon 10 in *Ae. tauschii* KU2001 (Fig. 2). The substitution G- > A is synonymous, but the deletion causes a frameshift and the potential formation of a defective protein (Fig. 2).

Several newly obtained sequences from *Ae. tauschii* accessions differed from allele *q-5D* of *Ae. tauschii* EU350482 and were closer to the D-genome sequences from hexaploids (Additional file 1: Table S3). The *Q* gene of the accessions K-1216 and TQ27 was similar to the *q-5D* of hexaploids, not only in substitutions within introns but also with respect to the G- > C transition in position 21 (Fig. 2).

Three accessions of *Ae. tauschii* (C21–5141, C21–5129, and KU-2074) are the most interesting among the analysed accessions, since their sequences of *q-5D* are highly similar or even identical in case of C21–5129 and KU-2074 to the *q-5D* of *T. aestivum* cv. Chinese Spring (JF701618) and *T. aestivum* cv. Renan (JF701615) (Additional file 1: Table S3). Furthermore, we analysed the most variable region of *q-5D*, including intron 9, exon 10 and partial 3’UTR, in 10 additional accessions of *Ae. tauschii* (Additional file 1: Table S1). Similarly to three accessions described above, the partial sequence of *q-5D* from *Ae. tauschii* K-1100 is identical to the *q-5D* of accessions of polyploid wheat (Additional file 1: Table S3). Moreover, the 3’UTR of the three novel sequences (MN206106, MN604289, MN604290) possess a CT-track of identical length to that described for the two well-studied cultivars of *T. aestivum*, directly confirming the participation of *Ae. tauschii* ssp. *strangulata* in the formation of hexaploid wheats.

Full-length *q-5S* gene sequences were obtained for four *Ae. speltoides* accessions (Fig. 2, Additional file 1: Table S4). The comparative analysis revealed that they are quite different from the *q-5B* sequences of polyploid wheats.

### Phylogeny of the *Q* genes sequences

**Fig. 3:**
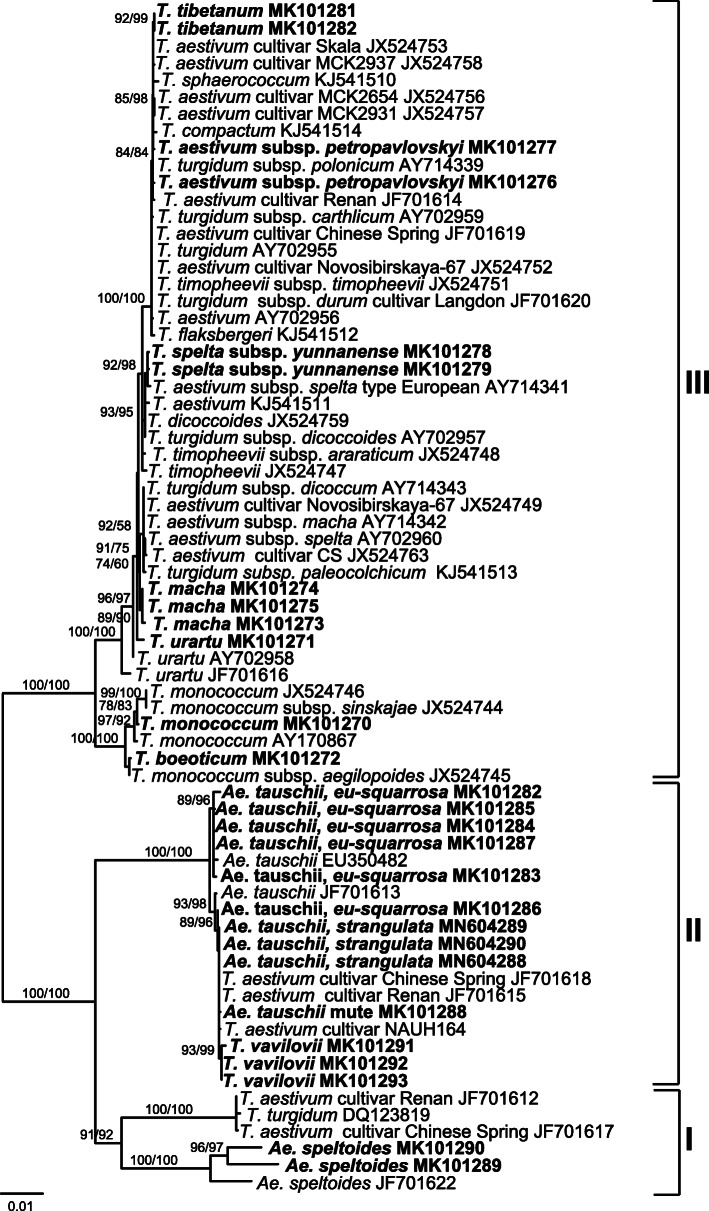
Maximum likelihood tree constructed on the basis of the full-length *Q* gene sequences from various *Triticum* and *Aegilops* species. Statistical support for the ML phylogeny was evaluated by ultra-fast bootstrap/aLRT. Nodes with statistical support over 50% are indicated. Clades are highlighted in different colours. Sequences identified in the present study are marked in bold

Phylogenetic analysis was performed for full-length DNA sequences of newly obtained and known *Q* gene sequences of different wheat species. In total, 70 sequences of A, B, D and S genome copies of the *Q* gene were used for the construction of a phylogenetic tree (Fig. 3). Three major clusters are present on the resultant tree. Cluster I consists of the *Q* gene sequences of the B genome of *Triticum* species and of the S genome of *Ae. speltoides*, where the latter form a separate group.

The D genome copies of the *Q* gene are included in two subclusters of cluster II. The *Ae. tauschii* species are present in both subclusters, i.e., the five sequences identified in the present study are clustered together with *q-5D* of the *T. aestivum* and *T. vavilovii* accessions (Fig. 3).

The third cluster has a clearly identifiable subcluster with *q-5A* sequences of the diploids *T. sinskajae*, *T. boeoticum* and *T. monococcum*. The remaining part of cluster III has a structure which is less clearly defined and is formed by both *q-5A* and *Q-5A* alleles from *T. urartu* and polyploid wheat species. Nevertheless, *Q-5A* sequences of *T. tibetanum* and *T. aestivum* ssp. *petropavlovskyi* were grouped together with tetraploid and hexaploid wheat species that possess the *Q-5A* allele. Within the cluster III, a branch with high support can be identified. This branch includes *T. tibetanum*, *T. aestivum* ssp. *petropavlovskyi, T. aestivum*, *T. polonicum*, *T. compactum* and *T. sphaerococcum*.

*Q* gene sequences of the Chinese endemic species *T. spelta* ssp. *yunnanense*, are located along with European *T. spelta*, which may confirm human participation in creating this species. The *T. macha* sequences form a separate branch within the group of *q-5A*-allele sequences. Sequences of *T. urartu* are located separately from polyploid wheats but do not form a single subcluster.

## Discussion

The origin of modern cultivated polyploid wheat species is a complicated and not fully elucidated history, which includes the emergence of species of the genus *Triticum* in both a natural way and as a result of human activity. Using a combination of bioinformatics and experimental methods of research usually allows the phylogenetic relationships of species within different taxa to be established. Unfortunately, species of the genus *Triticum* appeared about 10,000 years ago, and all the molecular markers used have a minimum number of nucleotide substitutions, which does not allow the phylogenetic relationships of all known species of this genus to be established in full. The standard chloroplast and mitochondrial markers allow the phylogeny of the genomes A, B, D and G to be determined, but do not allow the phylogenetic relationships of these species to be revealed [21, 22].

Therefore, an approach based on the analysis of various mutations occurring in the genes encoding the traits involved in domestication, most of which are transcription factors, was quite fruitful for identifying the emergence of species of the genus *Triticum* and establishing their phylogenetic relationships. This approach has been used in several studies, with a view to elucidating the evolution of diploid and tetraploid wheat species. In the present work, we used differences in the *Q-5A* and *Q-5D* genes to clarify the phylogeny of the genera *Triticum* and *Aegilops*.

It is known that the source of the A genome for all hexaploid species was *T. urartu*, and the source of both B and G genomes was *Ae. speltoides*, whose S genome is the ancestor for the B and G genomes in wheat. In all diploid wheat species, including the mutant *T. sinskajae*, only the variant V\GGC (329 valine and GGC motif within the miRNA172 binding site in the 10th exon) has been detected so far [present study; 1]. However, since accessions with fragile rachises and non fragile rachises are represented among diploid wheat species, the gene *Btr1-А* attracted attention of researchers [5, 6]. Pourkheirandish et al. (2018) identified that the non-synonymous change at the coding region of *Btr1-A* (A119T) determines the rachis fragility trait in diploid einkorn wheat species. The relationship between the *Btr1* and *Q* genes, as well as their joint contribution to the rachis fragility are poorly understood.

All hexaploids received the D genome from *Ae. tauschii*, and several studies have emphasized that the donor is *Ae. tauschii* ssp. *strangulata* (Eig) Zvelev but not *Ae. tauschii* ssp*. tauschii* [23–26]. In the present study, we established that several allelic variants of *q-5D* are present in the *Ae. tauschii* accessions. We have identified *q-5D* sequences from *Ae. tauschii* accessions (C21–5129, KU-2074, and K-1100) that are highly similar to those of hexaploid wheat accessions. The accession K-1100 is originated from Azerbaijan and the accessions C21–5129 and KU-2074 are originated from Iran. According to modern data, the crossing between the wild tetraploid wheat progenitor and *Ae. tauschii*, which led to the emergence of hexaploid wheat, occurred about 7000–7500 years ago in Central Asia [27]. This allows us to suggest that the accessions C21–5129, KU-2074, and K-1100 of *Ae. tauschii* ssp. *strangulata* could have been involved in the origin of hexaploid wheat species.

The *Q* gene is represented in the A genomes of hexaploids in three variants: V\GGC, I\GGT (329 isoleucine and GGT motif within the miRNA172 binding site in the 10th exon) and I\GAT (329 isoleucine and GAT motif within the miRNA172 binding site in the 10th exon). The original V\GGC variant of the species *T. macha* and *T. spelta ssp. indo-europeum* Vav. was directly obtained from tetraploid *T. dicoccum* and *T. spelta ssp. irano-asiaticum* Flaksb., which probably acquired it from the original tetraploid ancestor. *T. spelta ssp. yunnanense* stands apart, since this species carries a unique deletion in the *Q* sequence, which was found only in European *T. spelta* (AY714341). It should be noted that all the above-mentioned species contain the wild-type *Q* gene. Moreover, the Georgian endemic *T. macha* is located on the scheme separately from *T. spelta* ssp. *indo-europeum* and *T. spelta* ssp. *yunnanense*, due to unique mutations within the *q-5D* gene.

The remaining hexaploid species, including *T. aestivum*, have the I\GGT variant of the *Q* gene in the A genome, which does not originate from *T. dicoccoides*. It should be noted that another unique variant of the *Q* gene, I\GAT, was found in the genome of *T. aestivum* cv. Chinese Spring. As for *T. sphaerococcum* and *T. compactum*, *T. aestivum ssp. petropavlovskyi* probably originated from *T. aestivum*.

The results of Jiang et al. (2019) showed that the unique *Q-5A* allele of *T. tibetanum* originated from the *Q* gene of *T. aestivum*. The fragile rachis of *T. tibetanum* is a result of the re-acquisition of wild traits that occurred during de-domestication. According to our investigations, the *q-5D* gene sequences have two substitutions, which are described for the *T. macha* accessions only. The fragile rachis of the *T. aestivum* and the spelt spikes of *T. tibetanum* are probably determined by the gene *Tg*, and not by *Q* [9, 28]. *Tg* was described for hexaploid wheat species and localized on the short arm of the chromosome 2D, but until this gene has been molecularly cloned it is impossible to identify it’s relation to the gene *Q*.

**Fig. 4:**
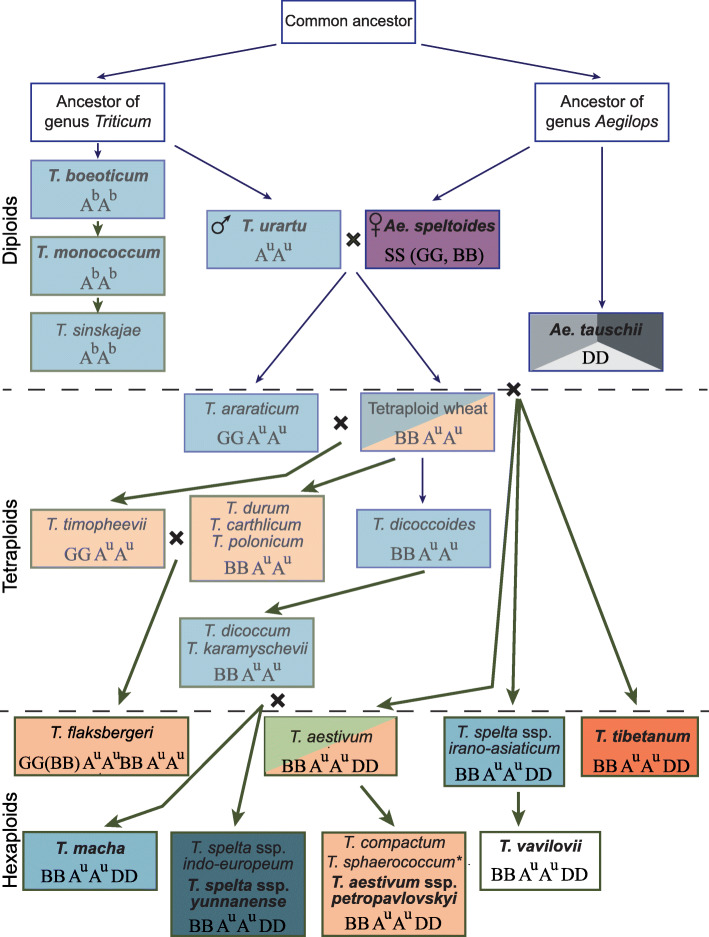
Predicted evolution of *Triticum* and *Aegilops* genera. Boxes are coloured according to the *Q* gene alleles detected in the accessions of the species (box colours match the colours in Fig. 2). Ancestral species are shown in white boxes. Blue arrows indicate natural selection and green arrows indicate artificial selection (domestication). ^*^Indicates the *Q* gene allele with isoleucine in the 329 position and SNPs 3125G, 3135G and 3139C. *T. karamyschevii* = *T. turgidum* ssp. *paleocolchicum*

Thus, our analysis allowed us to clarify the phylogenetic relationships of species of the genus *Triticum* (Fig. 4).

## Conclusions

The origin of cultivated wheat species and traits essential for domestication still remain an open question. Investigation of the genes which determine the traits involved in domestication of wheat allow to describe new alleles and are also promising for establishing the history of wheat’s origin. In this study, we studied the spelt factor gene, *Q,* for 15 accessions of wheat species, including four endemics, and 24 *Aegilops* accessions. Our analysis allowed us to clarify the phylogenetic relationships in the genus *Triticum*, namely show that the donor of the D genome for species of the genus *Triticum* is *Ae. tauschii* ssp. *strangulate*.

## Methods

### Plant material and growth conditions

Germplasm of di- and hexaploid wheat and *Aegilops* species, were obtained from 10 gene banks (Additional file 1: Table S1, Table S4). For each accession, 10 plants were grown under standard greenhouse conditions.

Threshability and rachis fragility traits were assessed using manual threshing. Spikes were classified as free threshing if their grains were surrounded by soft glumes which came off during threshing. Spikes with grains covered by tough glumes remaining adhered to the grain after the threshing were determined as non-free threshing. Non-fragile spikes were those which did not undergo self-dependent disarticulation, while fragile spikes displayed W- or B-type disarticulation. Spikes which separated from the whole straw without breaking into spikelets were a second type of fragile spike [29].

Spike shape has been determined visually in accordance with the following definitions: (1) the pyramidal spike with an elongated rachis and tenacious glumes is the spelt spike; (2) the short squareheaded parallel-sided spike is the normal spike; (3) the elongated spindle-shaped spike is the spelt-like spike. The spike shape has been confirmed by calculation of Flaksberger’s formula for spike density, D = 10(A - 1)/B, where (A - 1) is the number of spikelets per spike excluding the apical spikelet, and B is the length of the spike rachis in cm [30]. Calculated values for D displayed a high correlation with visual assessment of spike shape (see [31]).

### Total DNA extraction, PCR amplification, cloning and sequencing

Total DNA was isolated from 100 mg of leaves using the DNeasy Plant Mini Kit (QIAGEN) according to the manufacturer’s protocol. The *Q-5A* gene sequences of *Triticum* species and the *Q-5D* gene sequences of *Aegilops* species were PCR-amplified as six separate overlapping fragments, using primer pairs and PCR conditions as previously published [11]. In order to identify SNP (G- > A) within the miRNA172 binding site the partial sequences of the *q-5D* gene covering the region from intron 6 to exon 10 were amplified for hexaploid wheat accessions using the primer pair (Q-D1F, Q-D5R) designed by Zhao et al. [19]. PCR products were separated by agarose gel electrophoresis and purified using a QIAquick Gel Extraction Kit (QIAGEN). Purified PCR fragments were cloned into a pGEM®-T Easy vector using a pGEM-T Easy kit (Promega) and amplified using M13 primers prior to sequencing. Sequencing reactions were performed using 200 ng of the PCR product and a BigDye Terminator Kit on an ABI 3130xl Genetic Analyzer (Applied Biosystems) at the SB RAS Genomics Core Facility (http://www.niboch.nsc.ru/doku.php/corefacility). In total, 10 clones were sequenced for each fragment of *Q-5A* and *Q-5D* gene from all the studied accessions. *Q* gene sequences were deposited in GenBank.

### Sequence analysis

Multiple sequence alignment was performed using the MAFFT algorithm v. 7.397 [32] and then analysed in AliView 1.18.1 [33]. The p-distance was calculated using the Jukes-Cantor model in MEGA v. 5.10 [34]. A *Q* gene sequence region from the start codon to the stop codon (including introns) was used for phylogenetic analysis using the Maximum likelihood method in IQ-TREE v. 1.6.5 [35]. The best-fit evolutionary model (HKY + F + I) for the analysis according to the Bayesian information criterion (BIC), was evaluated using the ModelFinder algorithm implemented in the IQ-TREE package. Ultra-fast bootstrap and aLRT were used to estimate the statistical credibility of the obtained phylogenetic clades [36]. A *Q* gene sequences search was performed using BlastN and BlastP at https://www.ncbi.nlm.nih.gov/. OFR was predicted using the ORFfinder program, which was used for the search for open reading frames (ORFs) in the DNA sequence (https://www.ncbi.nlm.nih.gov/orffinder/). The miRNA binding sites were identified using the psRNATarget server [37].

## Supplementary information

**Additional file 1: Table S1.** Wheat species used in the study, their spike morphologies and *Q* gene alleles. **Table S2.** Spike shape determination for the wheat species used in the present study. **Table S3.** Variability of the *q-5D* gene sequences among *Aegilops* and *Triticum* species. **Table S4.** *Aegilops* species used in the study, their spike morphologies and *Q* gene alleles.

## Data Availability

The *Q* gene sequences are available in GenBank, with the accession numbers: MK101270-MK101302, MK101282-MK101290, MN206102-MN206113, MN604288-MN604290. Raw phylogenetic data were deposited at the FigShare (10.6084/m9.figshare.12562148). The datasets used and/or analysed during the current study available from the corresponding author on reasonable request.

## Abbreviations

BIC: Bayesian information criterion
ORFs: Open reading frames
PCR: Polymerase chain reaction
RNA: Ribonucleic acid
SNPs: Single-nucleotide polymorphisms
UTR: Untranslated region
