# Supplementary material for: Genetic variability of spelt factor gene in Triticum and Aegilops species
Source: BMC Plant Biol. 2020 Oct 14;20(Suppl 1):310. doi: 10.1186/s12870-020-02536-8 (PMC7556929; doi:10.1186/s12870-020-02536-8)
Supplement: Supplementary file 1 — Additional file 1: Table S1. Wheat species used in the study, their spike morphologies and Q gene alleles. Table S2. Spike shape determination for the wheat species used in the present study. Table S3. Variability of the q-5D gene sequences among Aegilops and Triticum species. Table S4. Aegilops species used in the study, their spike morphologies and Q gene alleles. [file 12870_2020_2536_MOESM1_ESM.doc]

**Additional file 1**

**Journal: BMC Plant Biology**

**Title: Genetic variability of spelt factor gene in *Triticum* and *Aegilops* species**

1. Research article
2. Valeriya Vavilova^1*^, Irina Konopatskaia^1^, Alexandr Blinov^1^, Elena Ya. Kondratenko^1^, Yuliya V. Kruchinina^1^, Nikolay P. Goncharov^1^
3. ^1^ Institute of Cytology and Genetics SB RAS, Novosibirsk, Russian Federation

###### **Correspondence:**

###### **Dr. Valeriya Vavilova**

1. ^*^ valeriya-vavilova@bionet.nsc.ru

**Table S1.** Wheat species used in the study, their spike morphology and *Q* gene alleles

| No. | Genome | Species^*^ | Accession | Spike morphology | *Q-5A* | *Q-5D* |
| --- | --- | --- | --- | --- | --- | --- |
| 1 | A^b^ | *Triticum monococcum* L. | PI 427927 | non free-threshing  fragile  spelt-like | *q-5A*  MK101270 | - |
| 2 | A^u^ | *T. urartu* Thum. ex Gandil. | Ig-44829 | non free-threshing  fragile  spelt-like | *q-5A*  MK101271 | - |
| 3 | A^b^ | *T. boeoticum* Boiss. | K-27148 | non free-threshing  fragile  spelt-like | *q-5A*  MK101272 | - |
| 4 | BA^u^D | *T. macha* Decapr. et Menabde | K-31689 | non free-threshing  fragile (second type)  spelt | *q-5A*  MK101273 | *q-5D*  MK101294 |
| 5 | BA^u^D | *T. macha* Decapr. et Menabde | K-28195 | non free-threshing  fragile (second type)  spelt | *q-5A*  MK101274 | *q-5D*  MK101295 |
| 6 | BA^u^D | *T. macha* Decapr. et Menabde | K-58671 | non free-threshing  fragile (second type)  spelt | *q-5A*  MK101275 | *q-5D*  MK101296 |

^*^ - species names are given according to (Dorofeev et al. 1979), Goncharov (2011)

*^**^- Q^t^* allele was described in the present study in parallel with Jiang et al. 2019.

**Table S1 (continued)**

| No. | Genome | Species^*^ | Accession | Spike morphology | *Q-5A* | *Q-5D* |
| --- | --- | --- | --- | --- | --- | --- |
| 7 | BA^u^D | *T. tibetanum* Shao | KU510 | non free-threshing  fragile (second type)  spelt | *Q^**^-5A*  MK101281 | *q-5D*  MK101301 |
| 8 | BA^u^D | *T*. *tibetanum* Shao | KU515 | non free-threshing  fragile (second type)  spelt | *Q^**^-5A*  MK101282 | *q-5D*  MK101302 |
| 9 | BA^u^D | *T. spelta* ssp. *yunnanense* (King ex S.L. Chen) N.P. Gontsch. | KU506 | non free-threshing  non fragile  spelt | *q-5A* (*=T. spelta* (AY714341))  MK101278 | *q-5D*  MK101299 |
| 10 | BA^u^D | *T. spelta* ssp. *yunnanense* (King ex S.L. Chen) N.P. Gontsch. | KU509 | non free-threshing  non fragile  spelt | *q-5A* (*=T. spelta* (AY714341))  MK101279 | *q-5D*  MK101300 |
| 11 | BA^u^D | *T. vavilovii* (Thum.) Jakibz. | Tri4630 | non free-threshing  non fragile  spelt | no sequence | *q-5D*  MK101291 |
| 12 | BA^u^D | *T. vavilovii* (Thum.) Jakibz. | Tri9416 | non free-threshing  non fragile  spelt | no sequence | *q-5D*  MK101292 |
| 13 | BA^u^D | *T. vavilovii* (Thum.) Jakibz. | Tri11556 | non free-threshing  non fragile  spelt | no sequence | *q-5D*  MK101293 |
| 14 | BA^u^D | *T. aestivum* ssp. *petropavlovskyi* (Udacz. et Migusch.) N.P. Gontsch. | KU502 | free-threshing  non fragile  spelt-like | *Q-5A*  MK101276 | *q-5D*  MK101297 |
| 15 | BA^u^D | *T. aestivum* ssp. *petropavlovskyi* (Udacz. Et Migusch.) N.P. Gontsch. | K-43351 | free-threshing  non fragile  spelt-like | *Q-5A*  MK101277 | *q-5D*  MK101298 |

^*^ - species names are given according to (Dorofeev et al. 1979), Goncharov (2011)

*^**^- Q^t^* allele was described in the present study in parallel with Jiang et al. 2019.

**Table S2.**  Spike shape determination for the wheat species used in the present study.

| **№** | **Species** | **Accession number** | **Spike length, cm** | **Spikelet number per spike** | **Spike density*** | **Spike shape** |
| --- | --- | --- | --- | --- | --- | --- |
| 1 | *T. monococcum* | T3, PI427927 | 4.4±1.02 | 17.1±6.56 | 36.5±8.40 | spelt-like |
| 2 | *T. urartu* | T15, Ig-44829 | 5±0.71 | 11±0.71 | 20±4.43 | spelt-like |
| 3 | *T. boeoticum* | K-27148 | 5.7±0.96 | 22.3±3.51 | 37.4±0.44 | spelt-like |
| 4 | *T. macha* | K-31689 | Information was obtained from database | | | Spelt |
| 5 | *T. macha* | K-28195 | 6.1±0.47 | 15.8±1 | 24.2±2.11 | Spelt |
| 6 | *T. macha* | K-58671 | 6±1.2 | 17.2±5.92 | 26.8±5.01 | Spelt |
| 7 | *T. tibetanum* | KU510 | 5.1±1.1 | 11.7±2 | 20.9±1.39 | Spelt |
| 8 | *T. tibetanum* | KU515 | 7±1.1 | 16.4±2 | 22.1±2.68 | Spelt |
| 9 | *T. spelta* ssp. *yunnanense* | KU506 | 8.3±1.1 | 15±2 | 16.9 | Spelt |
| 10 | *T. spelta* ssp. *yunnanense* | KU509 | Information was obtained from database | | | Spelt |
| 11 | *T. vavilovii* | Tri4630 | 8±1.1 | 13.2±2 | 15.2±1.5 | Spelt |
| 12 | *T. vavilovii* | Tri9416 | 5.8±1.1 | 13.3±2 | 21.1±1.4 | Spelt |
| 13 | *T. vavilovii* | Tri11556 | 7.2±1.1 | 13±2 | 16.7±1.94 | Spelt |
| 14 | *T. aestivum* ssp. *petropavlovskyi* | KU502 | 7.6±0.95 | 20.5±1.81 | 25.6±1.47 | spelt-like |
| 15 | *T. aestivum* ssp. *petropavlovskyi* | K-43351 | 7.8±1.1 | 14±2 | 16.7 | spelt-like |

*- Spike density was calculated using Flaksberger's formula.

**Table S3. Variability of the *q-5D* gene sequences among *Aegilops* and *Triticum* species.**

| Species name (GenBank accession #) | Exon 1 | | Intr1 | Intr 4 | Intr 5 | | | |  | Intr6 | | Intr7 | Intr 8 | Intr9 |
| --- | --- | --- | --- | --- | --- | --- | --- | --- | --- | --- | --- | --- | --- | --- |
|  | 21 | 72 | 485 | 905 | 1136 | 1414 | 1473 | 1479 | 1562 | 1713 | 1764-1767 | 1964 | 2116 | 2404 |
| *Ae. tauschii* EU350482 | G | G | C | C | C | C | T | T | C | G | CATG | T | G | A |
| ***Ae. tauschii* MK101283, MK101287** | C | G | C | C | C | C | C | C | C | G | CATG | T | G | A |
| ***Ae. tauschii* MK101284, MK101285** | C | G | C | C | C | C | T | T | C | G | CATG | T | G | A |
| ***Ae. tauschii* MN206104-MN206105, MN206107, MN206109- MN206113** | NI | NI | NI | NI | NI | NI | NI | NI | NI | NI | NI | NI | NI | A |
| ***Ae. tauschii* MK101282** | C | A | A | T | C | C | T | T | C | G | ---- | T | G | A |
| ***Ae. tauschii* MK101286, MK101288** | C | G | C | C | C | G | T | T | T | A | CATG | A | A | G |
| ***Ae. tauschii* MN604288** | C | G | C | C | C | G | T | T | T | A | CATG | A | A | G |
| ***Ae. tauschii* MN604289, MN604290** | C | G | C | C | C | G | T | T | T | A | CATG | A | A | G |
| ***Ae. tauschii* MN206106** | NI | NI | NI | NI | NI | NI | NI | NI | NI | NI | NI | NI | NI | G |
| ***Ae. tauschii* MN206108** | NI | NI | NI | NI | NI | NI | NI | NI | NI | NI | NI | NI | NI | G |
| *T. aestivum* cv. CS D JF701618 | C | G | C | C | C | G | T | T | T | A | CATG | A | A | G |
| *T. aestivum* cv. Renan JF701615 | C | G | C | C | C | G | T | T | T | A | CATG | A | A | G |
| ***T. macha* MK101294, MK101295, MK101296** | NI | NI | NI | NI | NI | NI | NI | NI | NI | A | CATG | A | A | G |
| ***T. tibetanum* MK101301, M101302** | NI | NI | NI | NI | NI | NI | NI | NI | NI | A | CATG | A | A | G |
| ***T. yunnanense* MK101299, MK101300** | NI | NI | NI | NI | NI | NI | NI | NI | NI | A | CATG | A | A | G |
| ***T. vavilovii* MK101292, MK10293** | C | G | C | C | T | G | T | T | T | A | CATG | A | A | G |
| ***T. vavilovii* MK101291** | C | G | C | C | T | G | T | T | T | A | CATG | A | A | G |
| ***T. petropavlovskyi* MK101297, MK101298** | NI | NI | NI | NI | NI | NI | NI | NI | NI | A | CATG | A | A | G |

**Table S3 (continued)**

| Species name (GenBank accession #) | Intr9 | | | | | | | | | | | | | |
| --- | --- | --- | --- | --- | --- | --- | --- | --- | --- | --- | --- | --- | --- | --- |
|  | 2404 | 2410 | 2538 | 2548 | 2578-2579 | 2607 | 2615 | 2628 | 2630 | 2650 | 2675 | 2678 | 2684 | 2726 |
| *Ae. tauschii* EU350482 | A | G | T | - | -- | - | - | - | A | - | - | - | T | T |
| ***Ae. tauschii* MK101283, MK101287** | A | G | T | A | TA | A | A | T | A | T | T | T | T | T |
| ***Ae. tauschii* MK101284, MK101285** | A | G | T | A | TA | A | A | T | A | T | T | T | T | T |
| ***Ae. tauschii* MN206104-MN206105, MN206107, MN206109- MN206113** | A | G | T | A | TA | A | A | T | A | T | T | T | T | T |
| ***Ae. tauschii* MK101282** | A | G | T | A | TA | A | A | T | A | T | T | T | C | T |
| ***Ae. tauschii* MK101286, MK101288** | G | G | T | A | TA | A | A | T | A | T | T | T | T | A |
| ***Ae. tauschii* MN604288** | G | G | T | A | TA | A | A | T | A | T | T | T | T | A |
| ***Ae. tauschii* MN604289, MN604290** | G | G | T | A | TA | A | A | T | A | T | T | T | T | A |
| ***Ae. tauschii* MN206106** | G | G | T | A | TA | A | A | T | A | T | T | T | T | A |
| ***Ae. tauschii* MN206108** | G | G | C | A | TA | A | A | T | A | T | T | T | T | A |
| *T. aestivum* cv. CS D JF701618 | G | G | T | A | TA | A | A | T | A | T | T | T | T | A |
| *T. aestivum* cv. Renan JF701615 | G | G | T | A | TA | A | A | T | A | T | T | T | T | A |
| ***T. macha* MK101294, MK101295, MK101296** | G | G | T | A | TA | A | A | T | G | T | T | T | T | A |
| ***T. tibetanum* MK101301, M101302** | G | G | T | A | TA | A | A | T | G | T | T | T | T | A |
| ***T. yunnanense* MK101299, MK101300** | G | G | T | A | TA | A | A | T | A | T | T | T | T | A |
| ***T. vavilovii* MK101292, MK10293** | G | G | T | A | TA | A | A | T | A | T | T | T | T | A |
| ***T. vavilovii* MK101291** | G | T |  | A | TA | A | A | T | A | T | T | T | T | A |
| ***T. petropavlovskyi* MK101297, MK101298** | G | G | T | A | TA | A | A | T | A | T | T | T | T | A |

**Table S3** (continued)

| Species name (GenBank accession #) | Intr9 | | | | | | | | | | **Exon 10** | | |
| --- | --- | --- | --- | --- | --- | --- | --- | --- | --- | --- | --- | --- | --- |
|  | 2769 | 2778 | 2800-2803 | 2829 | 2836 | 2840 | 2853 | 2991-2993 ins | 3010-3014 | 3015-3021 | 3097 | 3098 | 3117 |
| *Ae. tauschii* EU350482 | - | G | TTTC | - | G | C | A |  | TGCAG | GTGCAGG | C | G | T |
| ***Ae. tauschii* MK101283, MK101287** | C | G | TTTC | T | G | C | A |  | TGCAG | GTGCAGG | C | G | T |
| ***Ae. tauschii* MK101284, MK101285** | C | G | TTTC | T | G | C | A |  | TGCAG | GTGCAGG | C | G | T |
| ***Ae. tauschii* MN206104-MN206105, MN206107, MN206109- MN206113** | C | G | TTTC | T | G | C | A |  | TGCAG | GTGCAGG | C | G | T |
| ***Ae. tauschii* MK101282** | C | G | TTTC | T | G | C | G | CT | ----- | ------- | C | G | T |
| ***Ae. tauschii* MK101286, MK101288** | C | G | TTTC | T | A | C | A |  | TGCAG | GTGCAGG | C | G | T |
| ***Ae. tauschii* MN604288** | C | G | TTTC | T | A | C | A |  | TGCAG | GTGCAGG | C | G | T |
| ***Ae. tauschii* MN604289, MN604290** | C | G | TTTC | T | A | C | A |  | TGCAG | GTGCAGG | C | G | T |
| ***Ae. tauschii* MN206106** | C | G | TTTC | T | A | C | A |  | TGCAG | GTGCAGG | C | G | T |
| ***Ae. tauschii* MN206108** | C | G | TTTC | T | G | C | A |  | TGCAG | GTGCAGG | C | G | T |
| *T. aestivum* cv. CS D JF701618 | C | G | TTTC | T | A | C | A |  | TGCAG | GTGCAGG | C | G | T |
| *T. aestivum* cv. Renan JF701615 | C | G | TTTC | T | A | C | A |  | TGCAG | GTGCAGG | C | G | T |
| ***T. macha* MK101294, MK101295, MK101296** | C | A | TTTC | T | A | C | A |  | TGCAG | GTGCAGG | C | G | T |
| ***T. tibetanum* MK101301, M101302** | C | A | ---- | T | A | T | A |  | TGCAG | GTGCAGG | C | G | T |
| ***T. yunnanense* MK101299, MK101300** | C | G | TTTC | T | A | C | A |  | TGCAG | GTGCAGG | C | G | T |
| ***T. vavilovii* MK101292, MK10293** | C | G | TTTC | T | A | C | A |  | TGCAG | GTGCAGG | C | G | T |
| ***T. vavilovii* MK101291** | C | G | TTTC | T | A | C | A |  | TGCAG | GTGCAGG | C | T | T |
| ***T. petropavlovskyi* MK101297, MK101298** | C | G | TTTC | T | A | C | A |  | TGCAG | GTGCAGG | T | G | C |

**Table S3** (continued)

| Species name (GenBank accession #) | 3'UTR |
| --- | --- |
|  | 1 - 8 |
| *Ae. tauschii* EU350482 | -------- |
| ***Ae. tauschii* MK101283, MK101287** | --TCTCTC |
| ***Ae. tauschii* MK101284, MK101285** | -------- |
| ***Ae. tauschii* MN206104-MN206105, MN206107, MN206109- MN206113** | -------- |
| ***Ae. tauschii* MK101282** | -------- |
| ***Ae. tauschii* MK101286, MK101288** | -------- |
| ***Ae. tauschii* MN604288** | --TCTCTC |
| ***Ae. tauschii* MN604289, MN604290** | TCTCTCTC |
| ***Ae. tauschii* MN206106** | TCTCTCTC |
| ***Ae. tauschii* MN206108** | -------- |
| *T. aestivum* cv. CS D JF701618 | TCTCTCTC |
| *T. aestivum* cv. Renan JF701615 | TCTCTCTC |
| ***T. macha* MK101294, MK101295, MK101296** | NI |
| ***T. tibetanum* MK101301, M101302** | NI |
| ***T. yunnanense* MK101299, MK101300** | NI |
| ***T. vavilovii* MK101292, MK10293** | TCTCTCTC |
| ***T. vavilovii* MK101291** | TCTCTCTC |
| ***T. petropavlovskyi* MK101297, MK101298** | NI |

**Table S4.** *Aegilops* species used in the study, their spike morphology and *Q* gene alleles

| No. | Genome | Species | Accession | Spike morphology | *Q-5D/ Q-5S* |
| --- | --- | --- | --- | --- | --- |
| 1 | D | *Ae. tauschii* Coss., *eu-squarrosa* | KU2001 | non free-threshing, fragile, spelt | *q-5D*  MK101282 |
| 2 | D | *Ae. tauschii* Coss., *eu-squarrosa* | KU2009 | non free-threshing, fragile, spelt | *q-5D*  MK101283 |
| 3 | D | *Ae. tauschii* Coss., *eu-squarrosa* | KT120-16 | non free-threshing, fragile, spelt | *q-5D*  MK101284 |
| 4 | D | *Ae. tauschii* Coss., *eu-squarrosa* | C21-4030 | non free-threshing, fragile, spelt | *q-5D*  MK101285 |
| 5 | D | *Ae. tauschii* Coss., *eu-squarrosa* | K-1216 | non free-threshing, fragile, spelt | *q-5D*  MK101286 |
| 6 | D | *Ae. tauschii* Coss., *eu-squarrosa* | K-992 | non free-threshing, fragile, spelt | *q-5D*  MK101287 |
| 7 | D | *Ae. tauschii* Coss., *eu-squarrosa* | K-864 | non free-threshing, fragile, spelt | *q-5D*  MN206109 |
| 8 | D | *Ae. tauschii* Coss., *eu-squarrosa* | KU-2002 | non free-threshing, fragile, spelt | *q-5D*  MN206110 |
| 9 | D | *Ae. tauschii* Coss., *eu-squarrosa* | KU-2003 | non free-threshing, fragile, spelt | *q-5D*  MN206111 |
| 10 | D | *Ae. tauschii* Coss., *eu-squarrosa* | KU-20-6 | non free-threshing, fragile, spelt | *q-5D*  MN206112 |
| 11 | D | *Ae. tauschii* Coss., *eu-squarrosa* | K-608 | non free-threshing, fragile, spelt | *q-5D*  MN206104 |
| 12 | D | *Ae. tauschii* Coss., *eu-squarrosa* | C21-5144 | non free-threshing, fragile, spelt | *q-5D*  MN206113 |

**Table S4 (continued)**

| No. | Genome | Species | Accession | Spike morphology | *Q-5D/ Q-5S* |
| --- | --- | --- | --- | --- | --- |
| 13 | D | *Ae. tauschii* Coss., *eu-squarrosa* | K-912 | non free-threshing, fragile, spelt | *q-5D*  MN206105 |
| 14 | D | *Ae. tauschii* Coss., *eu-squarrosa* | K-1255 | non free-threshing, fragile, spelt | *q-5D*  MN206107 |
| 15 | D | *Ae. tauschii* Coss., *eu-squarrosa* | K-1330 | non free-threshing, fragile, spelt | *q-5D*  MN206108 |
| 16 | D | *Ae. tauschii* Coss., *strangulata* Eig | K-1100 | non free-threshing, fragile, spelt | *q-5D*  MN206106 |
| 17 | D | *Ae. tauschii* Coss., *strangulata* Eig | C21-5141 | non free-threshing, fragile, spelt | *q-5D*  MN604288 |
| 18 | D | *Ae. tauschii* Coss., *strangulata* Eig | C21-5129 | non free-threshing, fragile, spelt | *q-5D*  MN604289 |
| 19 | D | *Ae. tauschii* Coss., *strangulata* Eig | KU-2074 | non free-threshing, fragile, spelt | *q-5D*  MN604290 |
| 20 | D | *Ae. tauschii* Coss., mute | TQ27 | free-threshing, fragile, spelt | *q-5D*  MK101288 |
| 21 | S | *Ae. speltoides* Tausch | K-2366 | non free-threshing, fragile, spelt | *q-5S*  MK101289 |
| 22 | S | *Ae. speltoides* Tausch | K-1592 | non free-threshing, fragile, spelt | *q-5S*  MK101290 |
| 23 | S | *Ae. speltoides* Tausch | Ae-408 | non free-threshing, fragile, spelt | *q-5S*  MN206102 |
| 24 | S | *Ae. speltoides* Tausch | K-1591 | non free-threshing, fragile, spelt | *q-5S*  MN206103 |
